# Impact of transurethral resection of bladder tumors on sexual function and quality of life using ePROMs in patients with bladder cancer– a prospective cohort study

**DOI:** 10.1007/s00345-025-05726-x

**Published:** 2025-06-10

**Authors:** H.S. Menold, B. Gruene, J. Koenig, M. Lenhart, F. Waldbillig, K.F. Kowalewski, M.S. Michel, MC. Kriegmair, M. Neuberger, F. Wessels

**Affiliations:** 1 Department of Urology and Urosurgery, University Medical Center Mannheim, University of Heidelberg, Theodor-Kutzer-Ufer 1-3, 68167 Mannheim, Germany; 2 Clinic and Polyclinic for Child and Adolescent Psychiatry, Faculty of Medicine, University Hospital Cologne, Cologne, Germany; 3 DKFZ Hector Cancer Institute at the University Medical Center Mannheim, Heidelberg, Germany; 4 Division of Intelligent Systems and Robotics in Urology (ISRU), German Cancer Research Center (DKFZ), Heidelberg, Germany; 5 Department of Urology Munich-Planegg, Germeringer Str. 32, 82152 Planegg, Germany

**Keywords:** Bladder cancer; urinary bladder neoplasms, TURB, Patient-reported outcome measurements, Sexual function, Quality of life

## Abstract

**Introduction:**

The impact of transurethral resection of bladder tumors (TURBT) on patients’ quality of life (QoL) and sexual function is underrepresented in the literature. Therefore, this study aimed to evaluate sexual function and QoL following TURBT, using electronic patient reported outcome questionnaires (ePROMs).

**Methods:**

Patients undergoing TURBT were surveyed using a digital ePROM system (Heartbeat Medical). Sexual function and QoL were assessed using the Male/Female LUTS Sexual Function module (ICIQ-MLUTSsex/FLUTSsex) at 3, 6 and 12 months postoperatively and the EORTC QLQ-NMIBC 24. Linear mixed-effects models were used to identify influencing factors, adjusting for baseline. Repeated measures ANOVA tested differences in domain scores over time.

**Results:**

197 patients completed the survey, of whom 77% (*n* = 151) were men. Based on the ICIQ-MLUTSsex sexual function was significantly impaired at 3, 6 and 12-months postoperatively compared to baseline (*p* = 0.005; *p* = 0.004; *p* = 0.017). Age was the strongest factor for reduced male sexual function (ICIQ-MLUTSsex and QLQ-NMIBC24: *p* < 0.001). Subdomain analysis revealed negative effects on ejaculation (*p* = 0.044) and urinary symptoms (*p* = 0.031) up to 6 months. No differences were observed for the female population.

**Conclusion:**

TURBT may result in long-term impairment of sexual function in male patients, whereas no such effect was observed in female patients within this cohort.

**Supplementary Information:**

The online version contains supplementary material available at 10.1007/s00345-025-05726-x.

## Introduction

Transurethral resection of bladder tumors (TURBT) for treatment of bladder cancer (BC) is by far the most frequently performed procedure in urology. Almost 75% of bladder cancer cases are non-muscle invasive (NMIBC), encompassing both low-grade and high-grade tumors, and often requiring multiple TURBTs over time [1].

One of the most common concerns of cancer patients is maintaining quality of life (QoL) and minimizing treatment-related impact on psychosocial wellbeing and sexual function [2]. Compared to patients with other pelvic malignancies, this with BC report lower health-related quality of life (HRQoL) and in general higher rates of sexual dysfunction, especially in men [3]. In muscle-invasive bladder cancer (MIBC) the impact of QoL and sexual function have been well studied, especially in randomized controlled trials (RCT) e.g. after open and robotic-assisted cystectomy [4, 5]. In contrast, evidence on HRQoL and especially sexual function after TURBT for NMIBC remains limited. In particular, the generally low response rates for sensitive topics such as recording sexual function and consequently the collection of long-term data poses a challenge [6]. Although sexual issues are often part of question sets on QoL and are therefore included in QoL studies, a single self-reported questionnaire that covers the entire range of sexual health such as the ICIQ-MLUTSsex/FLUTSsex [7, 8] is usually not included in those studies [2]. Thus, the extent of the survey on this topic remains limited and superficial.

The use of validated electronic patient-reported outcome measurements (ePROMs) to record QoL and other treatment-specific outcome measurements is promising and has recently been shown to be feasible [9]. There is clear evidence that large-scale patient studies can improve patient outcomes, their HRQoL and satisfaction for better treatment control [10].

With the increasing focus on HRQoL in cancer patients, it is necessary to closely examine and assess sexual dysfunction peri-interventionally as well as long-term follow-up in order to raise awareness and consequently provide goal-directed treatment [11]. Therefore, the aim of this study was to evaluate sexual function and QoL for BC patients after TURBT using ICIQ-MLUTSsex/FLUTSsex and QLQ-NMIBC 24 as ePROMs.

## Materials and methods

### Study design

This study was performed at the Department of Urology at the University Medical Center Mannheim during 03/2019 until 03/2022 and was approved by the local ethics committee (2018–585 N-MA). Patients scheduled for a TURBT were prospectively screened for inclusion in this study. The following criteria had to be met for study inclusion: a minimum age of 18 years, ability to provide informed consent, sufficient German language skills to complete the questionnaires and access to an email account. Patients undergoing emergency interventions as well as patients with dementia or other cognitive impairments were excluded.

### ePROM system

The paperless ePROM system “heartbeat ONE” (heartbeat ONE by Heartbeat medical Solution GmbH, Berlin, Germany; https://heartbeat-med.com) was introduced at the institution in 2019. This system enables a digital patient survey that can be carried out on any web-enabled device. Furthermore, the system provides automatic scoring of the questionnaires. Patients willing to participate receive an email with a link to their survey, which can be answered via common web-browsers. In the context of our study, the patients included received an invitation to a preoperative baseline survey. Postoperatively, they received surveys at defined follow-up time points by email.

### Questionnaire and follow-up

The set of questionnaires included several questionnaires inquiring about aspects of HRQoL and sexual function. The time of completion for the different sets of questionnaires was approximately 10–15 min. The baseline survey consisted of the whole set of questionnaires and had to be completed preoperatively. To assess the sexual function, follow-up questionnaires were sent to all patients at similar time points (3, 6 and 12 months), namely the ICIQ-MLUTSsex/FLUTSsex [7, 8] and the QLQ- NMIBC24 [12], focusing on the domain of sexual function. Patients who had not yet reached the corresponding follow-up time point were excluded from the analysis for this time point.

#### Overview of patient-reported outcome measures

The ICIQ-MLUTSsex/FLUTSsex is a patient-completed questionnaire for detailed evaluation of gender-specific sexual matters associated with their lower urinary tract symptoms and impact on QoL. The questionnaire covers 4 items for males and for females (male items: erection, orgasm, pain/discomfort during ejaculation and impact of urinary symptoms; female items: pain/discomfort because of dry vagina, pain with sexual intercourse, impact of urinary symptoms, urine leakage with sexual intercourse) which can be rated from 0 to 3 (male) and 0–4 (female) depending to the extent. The overall score varies from 0 to 12 for males and 0–14 for females, with greater values indicating increasing problems with sexual function. Furthermore, the bladder cancer specific EORTC QLQ-NMIBC24 questionnaire was used to assess QoL and sexual function. The QLQ-NMIBC24 consists of functional domains, symptom domains and single item domains. All responses are linearly transformed from 0 to 100, with a higher score indicating more (severe) symptoms or problems or better function for the functional scales.

### Statistical analysis

A descriptive analysis was conducted to determine clinical characteristics and questionnaire responses across the sample population. Baseline characteristics are presented as median with interquartile range (IQR) or absolute and relative frequencies, respectively. Associations between clinical features and significant factors influencing sexual function for the two different questionnaires at the different follow-up time points postoperatively were calculated using mixed linear models. For the mixed linear model analyses, a full information maximum likelihood approach was applied in order to allow for the inclusion of all available data points without imputing missing values. Here, it was assumed that data were missing at random. For sensitivity analyses and to further evaluate the sexual function for the defined follow-up time points a repeated measurement ANOVA was performed. In this case, only participants with complete data across all timepoints were included. For Visualization, bar graphs were created using Graph Pad Prism Version 10.1.2. All statistical analyses were performed using IBM SPSS Version 29.0. Level of significance was set at 0.05.

## Results

### Study population characteristics

Overall *N* = 197 patients were invited to participate into the study. Baseline characteristics are shown in Table 1. Most patients were men (*n* = 151, 77%). Almost 70% had localized non-muscle-invasive tumors.

**Table 1:** Baseline characteristics

Variable	Male	Female
	*n* = 151	*n* = 46
	*n* (%)	*n* (%)
Age*	71 (66–76)	66 (60–74)
ICIQ Baseline*	3 (1–6)	5 (2–8)
QLQ-NMIBC 24 Baseline*	50 (17–67)	0 (0–33)
**Diagnosis of UCa**		
Yes	88 (58%)	20 (43%)
No	63 (42%)	26 (57%)
**Size of tumor**		
< 3 cm	42 (49%)	9 (47%)
≥ 3 cm	44 (51%)	10 (53%)
**Multifocal disease**		
unifocal	39 (45%)	12 (63%)
multifocal	47 (55%)	7 (37%)
**Repeat TUR-B**		
Yes	54 (63%)	14 (70%)
No	32 (37%)	6 (30%)
**Pathological tumor stage**		
PUNLUMP/Cis	10 (11%)	0
Ta	44 (50%)	13 (65%)
T1	16 (18%)	5 (25%)
T2	18 (21%)	2 (10%)
**EORTC stadium**		
low risk	13 (15%)	4 (19%)
intermediate risk	21 (24%)	9 (43%)
high risk	54 (61%)	8 (38%)

*Median (IQR-Interquartile Range), *n* (%); UCa– Urothelial carcinoma, TUR-B– Transurethral resection of bladder tumor, ICIQ- International Consultation on Incontinence Questionnaire, QLQ-Quality of Life

### Factors influencing sexual function

Table 2A and 2B provide an overview of parameters impacting gender-specific sexual function in men according to the mixed linear models. Age was a significant predictor for sexual function as measured by the ICIQ-MLUTSsex (*p* < 0.001). Furthermore, follow-up time points were a significant contributor for the ICIQ with baseline as reference (3-Mon *p* = 0.005; 6-Mon *p* = 0.004; 12-Mon: *p* = 0.017, Table 2A).

**Table 2A:** ICIQ-MLUTSsex

Variable	Estimator	SE	Significance	95% 95%CI
Age	0.095	0,27	**< 0**,**001**	0.042–0.149
Urothelial-Ca	1.120	2.02	0.58	-2.875–5.155
Size of tumor < / ≥ 3 cm	0.337	0.673	0.618	-0.997–1.671
Mutilfocality	0.364	0.673	0.590	-0.969–1.697
Baseline*	-	-	**-**	-
3-Mon FU	0.789	0.275	**0.005**	0.247–1.331
6-Mon FU	0.864	0.293	**0.004**	0.286–1.442
12-Mon FU	0.823	0.342	**0.017**	0.147–1.499

Linear mixed-effect models estimate for mean scores during 12-month follow-up

* reference level

**Table 2B:** QLQ-NMIBC

Variable	Estimator	SE	Significance	95%CI
Age	-1.107	0.312	**< 0.001**	-0.1723–0.490
Urothelial-Ca	-19.104	31.051	0.539	-80.195–41.897
Size of tumor < / ≥ 3 cm	-3.580	7.375	0.628	-18.142–10.981
Mulitfocality	-5.076	7.379	0.492	-19.646–9.495
Baseline*	-	-	-	-
3 Mon FU	5.310	5.374	0.324	-5.268–15.889
6 Mon FU	-2.626	5.813	0.652	-14.068–8.816
12 Mon FU	0.240	6.424	0.970	12.405–12.885

Linear mixed-effect models estimate for mean scores during 12-month follow-up

* reference level

Similarly, age was also a significant predictor for sexual function in men as measured with the QLQ (*p* < 0.001 Table 2B). All other parameters did not show statistical significance.

For the analyses in women, Table 2C and 2D depict the results of the mixed linear model. No significant predictors could be identified in our analysis.

**Table 2C:** ICIQ-FLUTSsex

Variable	Estimator	SE	Significance	95%CI
Age	-0.055	0.084	0.521	-0.236–0.125
Urothelial-Ca	0	0	-	-
Size of tumor < / ≥ 3 cm	-1.387	1.768	0.447	-5.224–2.450
Multifocality	1.418	2.168	0.525	-3.288–6.124
Baseline*				
3 Mon FU	1.174	1.312	0.380	-1.542–3.889
6 Mon FU	1.613	1.560	0.311	-1.598–4.824
12 Mon FU	-0.865	1.487	0.566	3.933–2.202

Linear mixed-effect models estimate for mean scores during 12-month follow-up

* reference level

**Table 2D:** QLQ-NMIBC

Variable	Estimator	SE	Significance	95%CI
Age	-0.584	0,478	0.234	-1.572–0.405
Urothelial-Ca	0	0	-	-
Size of tumor < / ≥ 3 cm	2.437	9.856	0.807	-18.107–22.980
Multifocality	-6.746	11.050	0.549	-29.919–16.427
Baseline*				
3 Mon FU	7.722	9.445	0.418	-11.343–26.786
6 Mon FU	-3.005	9.517	0.754	-22.223–16.213
12 Mon FU	0.40	10.082	0.997	-20.301–20.382

Linear mixed-effect models estimate for mean scores during 12-month follow-up

* reference level

The change in sexual function over time during follow up was analyzed using a repeated measurements ANOVA and visualized (see supplement Fig. 1A - B A - B). At 3- and 6-months follow-up, men reported worse sexual function scores, which gradually improved to the baseline scores again after 12 months. According to sexual function in the QLQ, mean scores were quite similar over time for male patients (supplementary Fig. 1B). For women, mean scores for sexual function postoperatively were worse, and then improved during the follow-up period, but these diffferences did not reach statistical significance. Mean sexual function scores were remarkably constant over time on the subdomain scores in the QLQ for women (supplementary Fig. 2B).

**Fig. 1:**
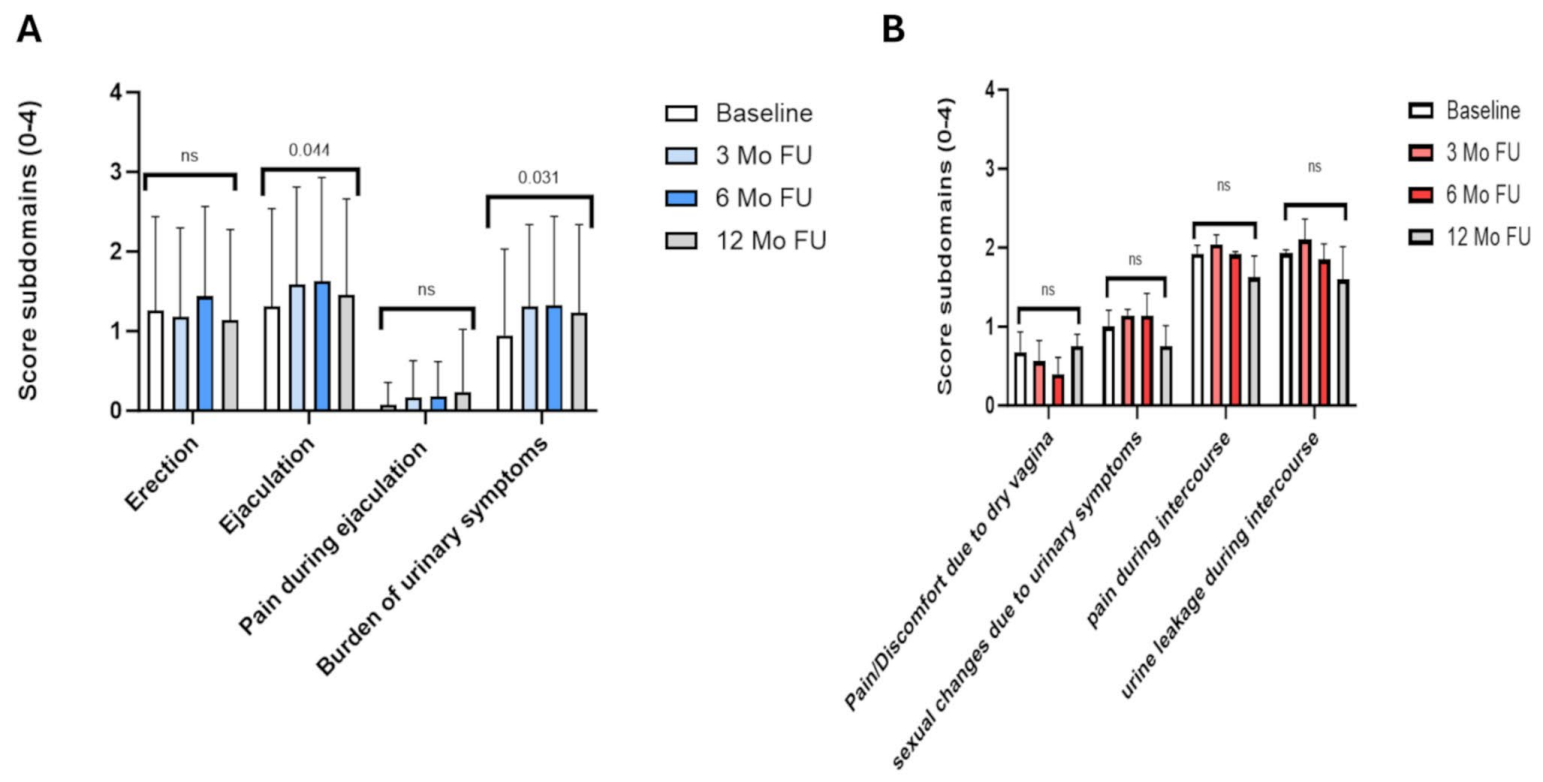
(**A**) Sexual function using the ICIQ sub domains for men. (**B**)Sexual function using the ICIQ sub domains for women. *higher scores meaning more (severe) problems for each domain, ns = not significant

### Subdomains of sexual function

We further explored subdomains (erection, ejaculation, pain during ejaculation and burden of urinary symptoms) of the ICIQ-FLUTSsex as part of our sensitivity analyses. While there were no changes for the domains “erection” and “pain during ejaculation”, we found that the domains “ejaculation” and “burden of urinary symptoms” were negatively impacted after TURBT but became gradually better again during follow-up (Fig. 1A).

For the ICIQ in women, the different subdomains (pain/discomfort due to dry vagina, sexual changes due to urinary symptoms, pain and urine leakage during intercourse) showed no significant differences in symptom scores over time (Fig. 1B).

## Discussion

In this single-center study we assessed QoL focusing on sexual function in BC patients undergoing TURBT, before and up to one year postoperatively, using ePROMs. Men’s sexual function was significantly impaired during 3,6 and 12 months follow-up compared to baseline, based on the ICIQ-MLUTSsex. Age showed to be the most influencing factor for decreased sexual function in men after TURBT. In the subdomain analysis of the ICIQ in men, ejaculation and burden of urinary symptoms were more pronounced up to 6 months postoperatively with a slight tendency to improve in the further follow-up period. For women, we could not identify significant parameters influencing sexual function after TURBT.

Although endoscopic treatment has less influence on the anatomical changes compared to radical open or minimally invasive treatment, we found significantly reduced sexual function scores postoperatively in men. Looking at the literature, there are several studies which confirmed a linkage between increasing age and impairment on sexual function, rather than disease stage or tumor characteristics, which is in line with our findings [3, 11, 13]. Moreover, additional comorbidities (e.g. cardiovascular disease) also play an important role on erectile dysfunction [14], along with other etiological factors such as psychological and emotional well-being as well as effects on relationships of patients and their partners, that were not investigated in our study [8].

As the first study assessing sexual function in NMIBC patients before and after surgery, a significantly reduced sexual function in men at the ICIQ-MLUTSsex was found 3,6, and 12 months postoperatively compared to baseline function. Similarly, Stav et al. reported at least a transient sexual dysfunction for treatment of NMIBC [15]. In a prospective study by Rogers et al., which analyzed sexual function after TURBT up to one year postoperatively, a slight improvement in sexual function was observed postoperatively, according to the QLQ-NMIBC [16]. In our study, a discrepancy between the ICIQ-MLUTSsex and the EORTC QLQ-NMIBC24 in detecting changes in sexual function was observed and merits further discussion. The ICIQ-MLUTSsex is a specialized and granular instrument developed specifically to assess sexual function in the context of lower urinary tract symptoms. It includes detailed subdomains addressing aspects such as ejaculation, erectile function, and urinary-related sexual concerns. In contrast, the EORTC QLQ-NMIBC24 contains only a limited number of items on sexual function and was primarily designed to assess broader aspects of health-related QoL in patients with NMIBC. This difference in focus may explain its lower sensitivity in detecting changes in overall QoL when only the sexual function domain varied over time, while other QoL domains remained stable.

Looking at the subdomains of the ICIQ-MLUTSsex, relevant problems with ejaculation and burden of urinary symptoms in male patients after TURBT were reported. This is in line with a large cross-sectional prospective survey from England that published similar results of significant ejaculatory problems and erectile difficulties after TURBT [2].

There is lack of evidence concerning the sexual function after TURBT in women. Research shows that women report lower levels of sexual activity compared to men across all age groups and that there is still a gender imbalance in sexual counseling [17, 18]. Moreover, women were less sexually active and had less sexual interest [2]. In our sample only a limited number of women completed the survey, reporting worse mean sexual function scores compared to men. Despite the already mentioned factors above, we assume that female NMIBC patients may have differing needs following their disease process, focusing more on emotional and psychological factors, without being able to draw any further conclusions on our population.

Given the physiological and psychological complexity associated with sexual health among NMIBC patients, it is necessary to provide education about the effect of treatment on sexuality and encouragement of communication about sexual concerns considering multidisciplinary care [11]. Overall, although patients undergoing TURBT can be reassured regarding potential negative effects on HRQoL due to generally short-living symptoms, a prolonged postoperative recovery for sexual function in men must be discussed with the patients [19].

This study comes with several limitations, which must be considered. Due to a single center study, one of the main limitations is the relatively small sample size of only two hundred patients included. Furthermore, it must be taken into account, that the ICIQ-MLUTSsex/FLUTSsex is not a cancer-specific validated questionnaire. Moreover, with regard to the possible influencing factors on sexual function, comorbidities were not further analyzed. Nonetheless, despite these limitations the data are valuable for clinicians for different reasons:

- Only few studies have assessed sexual function after TURBT.
- Lack of data on sexual function in NMIBC patients before and after TURBT in comparison.

In conclusion, TURBT impacts the sexual function of men during postoperative course of one year. This must be taken into consideration to the pre-op counselling process and shared decision making before undergoing TURBT, and even more importantly it needs to be addressed in postoperative care. For women, no clear impact could be shown within our trial.

## Electronic Supplementary Material

Below is the link to the electronic supplementary material.

Supplementary Material 1

## Data Availability

No datasets were generated or analysed during the current study.
