# Supplementary material for: Impact of transurethral resection of bladder tumors on sexual function and quality of life using ePROMs in patients with bladder cancer– a prospective cohort study
Source: World J Urol. 2025 Jun 10;43(1):370. doi: 10.1007/s00345-025-05726-x (PMC12152061; doi:10.1007/s00345-025-05726-x)
Supplement: Supplementary file 1 — Supplementary Material 1 [file 345_2025_5726_MOESM1_ESM.docx]

## Supplementary File

**Figure 1A:** Development of sexual function based on ICIQ-MLUTS during follow-up period of 12 months

**Figure 1B:** Development of sexual function based on MQLQ- NMIBC during follow-up period of 12 months

**Figure 2A:** Development of sexual function based on ICIQ-FLUTS during follow-up period of 12 months

**Figure 2B:** Development of sexual function based on FQLQ-NMIBC during follow-up period of 12 months
